# Intracranial Atherosclerotic Plaque Characteristics and Burden Associated With Recurrent Acute Stroke: A 3D Quantitative Vessel Wall MRI Study

**DOI:** 10.3389/fnagi.2021.706544

**Published:** 2021-07-28

**Authors:** Beibei Sun, Lingling Wang, Xiao Li, Jin Zhang, Jianjian Zhang, Xiaosheng Liu, Hengqu Wu, Mahmud Mossa-Basha, Jianrong Xu, Bing Zhao, Huilin Zhao, Yan Zhou, Chengcheng Zhu

**Affiliations:** ^1^Department of Radiology, Renji Hospital, School of Medicine, Shanghai Jiaotong University, Shanghai, China; ^2^Department of Nuclear Medicine, Fudan University Shanghai Cancer Center, Shanghai, China; ^3^Department of Neurology, Renji Hospital, School of Medicine, Shanghai Jiaotong University, Shanghai, China; ^4^Department of Radiology, University of Washington, Seattle, WA, United States; ^5^Department of Neurosurgery, Renji Hospital, School of Medicine, Shanghai Jiaotong University, Shanghai, China

**Keywords:** intracranial artery, atherosclerosis, recurrent acute stroke, culprit plaque, 3D high-resolution vessel wall MR imaging

## Abstract

**Background:** Intracranial atherosclerotic disease (ICAD) tends to affect multiple arterial segments, and previous studies rarely performed a comprehensive plaque analysis of the entire circle of Willis for the evaluation of recurrent stroke risk. We aimed to investigate the features of circle of Willis ICAD on 3D magnetic resonance vessel wall imaging (MR-VWI) and their relationships with recurrent acute stroke.

**Methods:** Patients with either acute ischemic stroke (within 4 weeks after stroke) or chronic ischemic stroke (after 3 months of stroke) due to intracranial atherosclerotic plaque underwent 3D contrast-enhanced MR-VWI covering major cerebral arteries. Participants were divided into three groups: first-time acute stroke, recurrent acute stroke, and chronic stroke. Culprit plaque (defined as the only lesion or the most stenotic lesion when multiple plaques were present within the same vascular territory of the stroke) and non-culprit plaque characteristics, including total plaque number, plaque thickness, plaque area, plaque burden (calculated as plaque area divided by outer wall area), enhancement ratio (ER), eccentricity, and stenosis, were measured and compared across the three groups. Associations between plaque characteristics and recurrent acute stroke were investigated by multivariate analysis.

**Results:** A total of 176 participants (aged 61 ± 10 years, 109 men) with 702 intracranial plaques were included in this study. There were 80 patients with first-time acute stroke, 42 patients with recurrent acute stroke, and 54 patients with chronic stroke. More intracranial plaques were found per patient in the recurrent acute stroke group than in the first-time acute stroke or chronic stroke group (5.19 ± 1.90 vs. 3.71 ± 1.96 and 3.46 ± 1.33, *p* < 0.001). Patients in the recurrent acute stroke group had greater culprit plaque burden (*p* < 0.001) and higher culprit ER (*p* < 0.001) than the other two groups. After adjustment of clinical demographic factors, in multivariate analysis, coronary artery disease (CAD) (odds ratio, *OR* = 4.61; *p* = 0.035), total plaque number (*OR* = 1.54; *p* = 0.003), culprit plaque ER (*OR* = 2.50; *p* = 0.036), and culprit plaque burden (*OR* per 10% increment = 2.44; *p* = 0.010) were all independently associated with recurrent acute stroke compared to the first-time acute stroke.

**Conclusion:** Increased intracranial atherosclerotic plaque number, higher culprit plaque ER, greater culprit plaque burden, and CAD are independently associated with recurrent acute stroke.

## Background

Intracranial atherosclerotic disease (ICAD) is a common cause of ischemic stroke in Asians (Holmstedt et al., 2013; Wang et al., 2014) and is associated with a high risk of stroke recurrence (as high as 10–24% annually) (Mazighi et al., 2006). Determination of the factors associated with stroke recurrence is critical for the secondary prevention of stroke. Previous studies suggest that several vascular risk factors such as high blood pressure, diabetes, the circle of Willis variations, and previous coronary artery disease (CAD) are well-known risk factors for recurrent stroke (Holmstedt et al., 2013; Wang et al., 2014; Kang et al., 2016). Patients with severe symptomatic intracranial stenosis (70–99% of the diameter of a major intracranial artery) are at particularly high risk for recurrent stroke in the territory of the stenotic artery (5–23% at 1 year) despite under standard medication (Kasner et al., 2006; Zaidat et al., 2008; Chimowitz et al., 2011; Wang et al., 2014). Besides, ICAD is a systemic disease and can affect multiple arterial segments and vascular beds (Hoshino et al., 2018). Many large prospective studies have documented that the presence of multiple intracranial atherosclerotic stenoses (ICAS) or concurrent extracranial atherosclerosis, on computed tomography angiography (CTA) (Lau et al., 2013), magnetic resonance angiography (MRA) (Man et al., 2009; Wang et al., 2014; Sun et al., 2018), or digital subtraction angiography (DSA) (Zhao et al., 2018), was an independent predictor of stroke recurrence. Coexisting cerebrovascular atherosclerosis involving both intracranial and extracranial arterial beds is an independent predictor for subsequent vascular events (including ischemic stroke) (Li et al., 2020).

During the past decades, the development of vessel wall MRI has enabled the evaluation of intracranial plaque features *in vivo*, and recently, three-dimensional (3D) MR vessel wall imaging (MR-VWI) allows for the evaluation of plaque in the entire Circle of Willis with high isotropic resolution (Qiao et al., 2011; Xie et al., 2016). The intracranial plaque enhancement detected by gadolinium-contrasted MRI is associated with vasa vasorum, which is possibly a surrogate marker of vessel wall inflammation (Portanova et al., 2013), and it was associated with stroke risk (Rudd and Fayad, 2008; Qiao et al., 2014). There were a few studies that investigated the association between plaque features using MR-VWI and recurrent stroke risk and found that contrast enhancement and plaque burden were the potential factors associated with recurrent stroke (Kim et al., 2016; Ran et al., 2020; Shi et al., 2021). However, these studies were limited in the use of two-dimensional (2D) imaging, only one single segment [middle cerebral artery (MCA) stenosis], and only in patients with anterior circulation stroke.

We hypothesize that the number of intracranial plaques and vulnerable plaque features revealed on 3D contrast-enhanced MR-VWI are associated with stroke recurrence. This study sought to investigate whether these features are associated with patients with recurrent acute stroke in both the anterior and posterior circulation.

## Methods

### Study Population

This was a prospective, cross-sectional study, and the study protocol was approved by the institutional review board, and informed written consent was obtained from all patients. Patients with ischemic stroke symptoms prospectively and consecutively underwent 3D contrast-enhanced MR-VWI covering major cerebral arteries from October 2017 to December 2020, and we reviewed these images in 1 week after scans (from October 2017 to January 2021). Inclusion criteria for this study were as follows: (1) patients with intracranial arterial stenosis detected on MRA or CTA (including bilateral C6-7 segment of internal carotid artery, A1-2 segment of anterior cerebral artery, M1-2 segment of MCA, V4 segment of vertebral artery, P1-2 segment of posterior cerebral artery and basilar artery); (2) ischemic infarct confirmed by diffusion-weighted imaging (DWI) for the acute group, and chronic infarct was identified from T2-fluid attenuated inversion recovery imaging (FLAIR) and DWI; and (3) stroke etiology determined to be ICAS by the identification of intracranial artery plaque on 3D MR-VWI. Exclusion criteria were as follows: (1) intracranial artery occlusion; (2) coexistence of >50% stenosis or unstable plaques (presence of at least three of the following features: calcification, hemorrhage, superficial irregularity, and being lipid-rich) of the ipsilateral extracranial carotid artery detected by imaging (ultrasound, MRA, CTA, or DSA); (3) evidence of cardioembolic source ischemic stroke (recent myocardial infarction within 3 weeks, atrial fibrillation or flutter, evidence of cardiac or valvular thrombus on echocardiography, or other imaging; (4) clinical evidence of the presence of vasculopathy other than atherosclerosis (e.g., vasculitis, reversible cerebral vasoconstriction syndrome or other vasospastic processes, Moyamoya disease, or dissection); (5) degraded image quality of 3D MR-VWI that limited accurate delineation of the artery boundaries for quantitative analysis; and (6) patients with subacute stroke (time from onset:1–3 months). Recurrent acute stroke events were defined as new neurological deficits that fit the definition of acute ischemic stroke occurring 24 h after the incident strokes in the same vascular territory and were not attributable to cerebral edema, mass effect, or hemorrhagic transformation (Coull and Rothwell, 2004). Participants were divided into three groups: first-time acute stroke, recurrent acute stroke, and chronic stroke.

Clinical information including age, gender, body mass index (BMI), vascular risk factors (hypertension, hyperlipidemia, diabetes mellitus, smoking status, CAD), and relevant medications (statins and antiplatelet agents) was recorded for each patient. Hypertension was defined as a systolic blood pressure ≥140 mmHg, a diastolic blood pressure ≥90 mmHg, or current treatment with antihypertensive agents. Dyslipidemia was defined as TC/HDL-C ratio ≥5, measured LDL-C ≥ 3.5 mmol/l, or taking lipid-modifying medications. Smoking status was assessed at the time of the ischemic event, and the patients were dichotomized into two groups: current smoker (defined as a patient who had smoked continuously for 6 months with ≥1 cigarette per day) (Wang et al., 2014) or not a current smoker. Clinical history of previous strokes was recorded for patients in the recurrent acute stroke group.

### 3D MR-VWI Protocol

All the subjects underwent brain MRI on a 3.0T whole-body scanner (Philips Ingenia, Philips Healthcare, Best, The Netherlands). The MR protocol included three-dimensional time-of-flight (3D-TOF) MRA and 3D MR-VWI. The imaging parameters of these sequences were as follows: (1) 3D-TOF MRA: TR/TE: 23/3.5 ms, flip angle 18°, field of view 199 mm × 199 mm; slice thickness 1.2 mm, and acquisition matrix 500 × 332; (2) T1WI volume isotropic turbo spin-echo acquisition (T1-VISTA): coronal imaging orientation, TR/TE 500/25 ms, TSE factor 45, with variable flip angle, number of slices 120, FOV 230 m × 250 mm × 50 mm, voxel size 0.6 × 0.6 × 0.6 mm, scan time 7 min 10 s; (3) Simultaneous Non-contrast Angiography and intraPlaque hemorrhage (SNAP): FFE, TR/TE 9.9/4.8 ms, flip angle 11/5°. Post-contrast T1-VISTA images were acquired 5 min after intravenous gadolinium contrast agent (Bayer Schering Pharma AG, Germany) injection (with a dose of 0.1 mmol/kg, at a rate of 1.5 ml/s) with similar imaging parameters as pre-contrast. DWI and T2-FLAIR were used for infarct identification.

### Image Analysis

Evaluation of MR-VWI was conducted by two experienced radiologists (XL and LW, each with 5 years of experience in intracranial artery imaging) who were blinded to patient clinical information and performed a quantitative evaluation of plaque characteristics. A subgroup of 30 cases was randomly selected from the studied population (10 cases in the first-time acute stroke group, 10 cases in the recurrent acute stroke group, and 10 cases in the chronic stroke group) for a reproducibility study. Two readers (XL and LW) independently performed all measurements on the 30 cases for the evaluation of inter- and intra-reader agreement. One reviewer (XL) re-evaluated the same 30 cases independently after 2 months. Image quality rating was assigned using a four-point scale (1, poor; 2, marginal; 3, good; and 4, excellent) depending on the overall signal-to-noise ratio and the clarity of the vessel wall boundaries (Zhao et al., 2013), and the MR images with image quality ≥3 were qualified for analysis. All atherosclerotic plaques on pre-contrast MR-VWI were then identified using a previously reported definition, that is, the presence of focal wall thickening (Qiao et al., 2014). Lumen and outer wall boundaries were manually segmented on both pre-contrast and post-contrast T1-VISTA, using medical imaging viewer software (Vue PACS Livewire, Carestream, Rochester, NY, United States). The contouring of the plaques in each patient took 10–20 min depending on the number of plaques per patient.

Plaque quantitative parameters assessed included plaque thickness, plaque length, plaque area, plaque burden, plaque enhancement ratio (ER) and enhancement score, and luminal stenotic rate. Qualitative parameters included eccentricity, enhancement grade, and intraplaque hemorrhage (IPH). Luminal stenotic rate, plaque length, plaque burden, and ER were measured on the reconstructed pre-contrast and post-contrast images at the site of the most stenotic lesion or the most apparent wall thickening for each participant. The degree of stenosis was measured on black blood T1-VISTA images using WASID criteria:

$Degree\;of\;stenosis=\left(1-\frac{D_{stenosis}}{D_{normal}}\right)\times 100\%$ (Samuels et al., 2000), where D_*stenosis*_ denotes the diameter of the artery at the site of the most severe stenosis and D_*normal*_ denotes the diameter of the proximal normal artery. The use of 3D MR-VWI for intracranial stenosis measurements has been validated against luminal imaging techniques with the excellent agreement (Tian et al., 2021). The plaque burden was calculated as:

$$\begin{array}{l} Plaque\;burden=\frac{total\;wall\;area-lumen\;area}{total\;wall\;area}\times 100\% \end{array}$$

(Qiao et al., 2016).

Diagram for the measurements of plaque morphology is shown in Supplementary Figure 1.

The ER was measured at the slice of greatest enhancement, using adjacent gray matter (in a region of ~15 mm^2^) to normalize the signal intensity (SI). The ER was calculated as $Enhancement\;ratio=\frac{\frac{{SI\text{\_}Plaque}_{postcontrast}}{{SI\text{\_}gray\;matter}_{postcontrast}}-\frac{{SI\text{\_}Plaque}_{precontrast}}{{SI\text{\_}gray\;matter}_{precontrast}}}{\frac{{SI\text{\_}Plaque}_{precontrast}}{{SI\text{\_}gray\;matter}_{precontrast}}}\;$ (Shi et al., 2018) (SI short for signal intensity). Plaque enhancement grade was classified into three grades on post-contrast T1-VISTA images by using published criteria (Qiao et al., 2014; Hartman et al., 2019): grade 0, no enhancement, defined as the SI of plaque was similar to that of adjacent normal vessel wall; grade 1, mild to moderate enhancement, defined as the SI of plaque was lower than that of the pituitary infundibulum but higher than that of adjacent normal vessel wall; and grade 2, significant enhancement, defined as the SI of plaque was similar to or greater than that of the pituitary infundibulum. The plaque enhancement score of each patient was calculated by summing all enhancement grades of all plaques in intracranial arteries (Cui et al., 2020).

Eccentricity was defined as a localized plaque surrounding <75% of the vessel wall (Chung et al., 2012). The presence of IPH was defined as >150% signal relative to muscles within the covered anatomy on pre-contrast T1-weighted images (Zhu C. et al., 2018).

The culprit plaque was defined as (a) the only lesion within the vascular territory of the stroke or (b) the most stenotic lesion when multiple plaques were present within the same vascular territory of the stroke (Qiao et al., 2014). The other plaques that were not culprit plaque were defined as non-culprit.

### Statistical Analysis

Quantitative and categorical data are presented as mean ± SD or median (range) and counts or percentages, respectively. Comparisons between groups were made using chi-square/Fisher's test for categorical data and the Mann–Whitney *U*-test or Student's *t*-test for continuous data. The inter- and intra-observer agreement was calculated through the Cohen κ value for categorical data, and by intraclass correlation coefficient (ICC) and Bland–Altman analysis for continuous data. A value of κ and ICC > 0.80 was used to indicate an excellent agreement. Parameters with a *p* < 0.1 from univariate analysis were included in the following multivariate logistic regression analysis, which was used to determine the independent factors associated with recurrent stroke. Multicollinearity testing was performed using variance inflation factor (VIF) as an evaluation standard. A VIF that equals 1 indicates no multicollinearity among factors; a VIF between 1 and 5 indicates moderate collinearity; a VIF between 5 and 10 indicates a high correlation that may be problematic; a VIF > 10 indicates a significant correlation leading to unreliability of the regression analysis (Kim, 2019). All tests were two-tailed, and *p* < 0.05 were considered significant. The receiver-operating characteristic (ROC) curves were plotted for parameters with independent significance, and area under curves (AUC) were calculated. SPSS (Statistics version 22) was used for data analysis. MedCalc version 19.1 was used for drawing ROC curves.

## Results

### Characteristics of Patients

**Figure 1:**
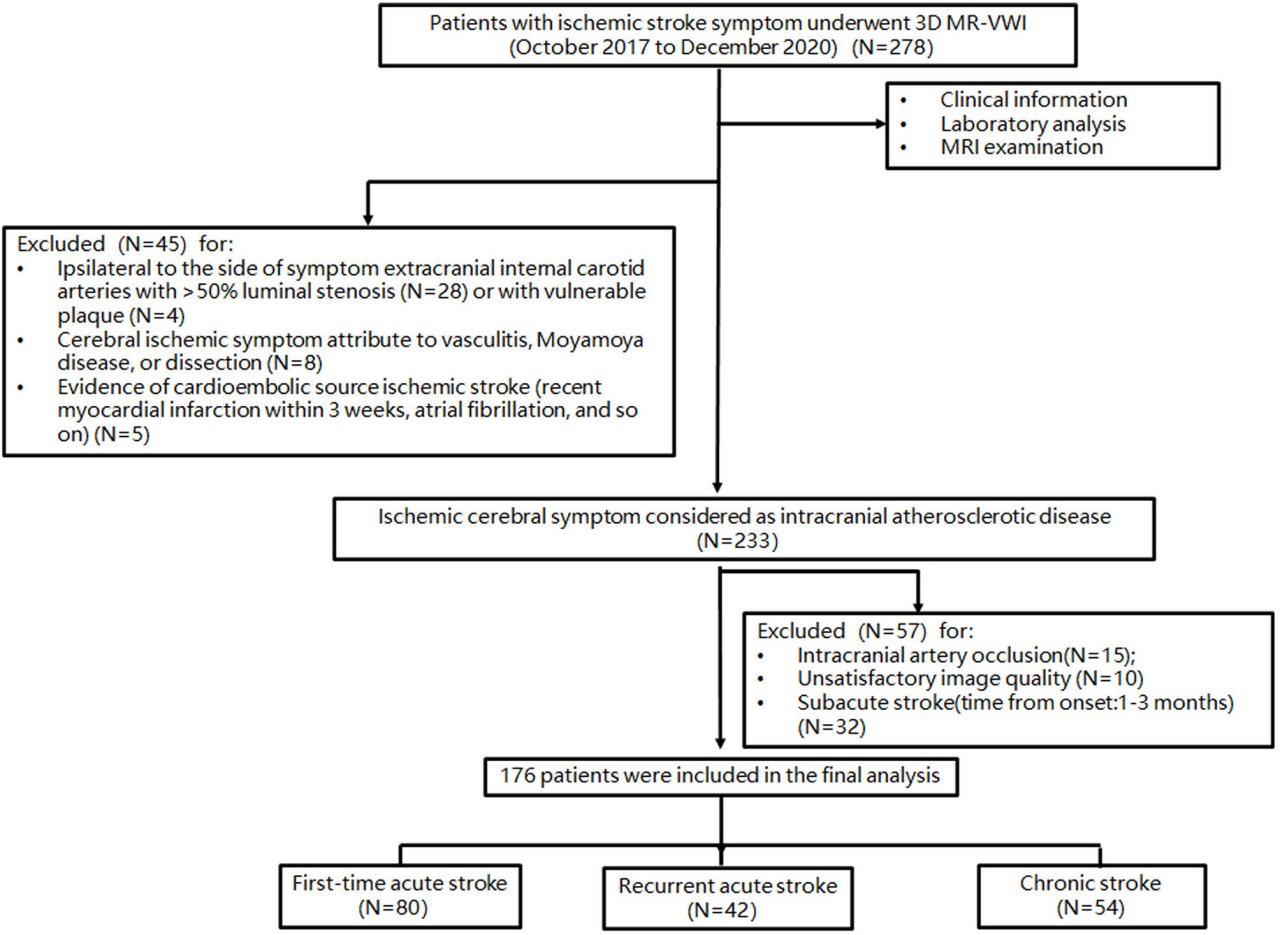
Patient selection flow chart.

The patient selection flowchart is shown in Figure 1. From October 2017 to December 2020, 278 patients met the inclusion criteria. After the exclusion of 102 patients, 176 patients [aged 61 ± 10 years; 109 (61.9%) men] were included in the final analysis. The demographic information of patients is listed in Table 1. Of these patients, 132 (75%) had hypertension, 69 (39.2%) had diabetes, 51 (28.9%) had dyslipidemia, 44 (25%) had family history of cerebro-cardiovascular disease, 19 (10.8%) had CAD, and 32 (18.2%) were current smokers at the time of imaging. There were 80 first-time acute stroke patients, 42 recurrent acute stroke patients, and 54 chronic stroke patients. Recurrent acute stroke occurred with a mean of 12.6 ± 23.9 months (median 3.5 months, interquartile range 1–10.5 months) after the previous events. The age of chronic stroke patients was older than that of the first-time acute stroke patients (*p* = 0.025). The prevalence of CAD of recurrent acute stroke patients was higher than that of the first-time acute stroke patients (*p* = 0.022). Pre-admission statin and aspirin use was more common in the recurrent acute stroke group than in the other two groups, while dual antiplatelet use was more common in the recurrent acute stroke group compared with the chronic stroke group, but no different from the first-time acute stroke patients. No significant difference was observed in other clinical characteristics among the three groups.

**Table 1:** Demographic information of the study population.

	**Group 1, patients with first-time acute stroke (** ***N*** **= 80)**	**Group 2, patients with recurrent acute stroke (** ***N*** **= 42)**	**Group 3, patients with chronic stroke (** ***N*** **= 54)**	***p^a^*** **-value**	**Between-group** ***p*** ^**b**^ **values: group 1 vs. 2; group 2 vs. 3, group 1 vs. 3**
Male, *n* (%)	53 (66.3%)	24 (57.1%)	32 (59.3%)	0.547	0.322, 0.835, 0.410
Age, year	59.45 ± 9.85	60.64 ± 9.40	63.32 ± 9.37	0.075	0.515, 0.178, 0.025
BMI, kg/m^2^	24.60 ± 2.89	24.58 ± 2.34	24.68 ± 2.21	0.978	0.957, 0.845, 0.866
Interval between symptom and MRI scan^#^	20d (2d−30d)	15d (2d−30d)	6M (3M−48M)	NA	0.158^†^
Baseline NIHSS^#^	2 (0–8)	2.5 (0–6)	2 (0–8)	0.107	0.587, 0.051, 0.090
**Risk factors**					
Current smoking, *n* (%)	15 (18.8%)	10 (23.8%)	7 (13.0%)	0.387	0.511, 0.188, 0.375
Alcohol, *n* (%)	7 (8.8%)	7(16.7%)	3(5.6%)	0.175	0.235, 0.098, 0.739
Family history of CVD, *n* (%)	18 (22.5%)	10 (23.8%)	16 (29.6%)	0.633	0.870, 0.949, 0.352
DM, *n* (%)	36 (45.0%)	17 (40.5%)	16 (29.6%)	0.199	0.632, 0.524, 0.073
CAD, *n* (%)	4 (5.0%)	8 (19.0%)	7 (13.0%)	0.042	0.022, 0.415, 0.118
Hypertension, *n* (%)	56 (70.0%)	34 (81.0%)	42 (77.8%)	0.353	0.191, 0.704, 0.319
SBP, mmHg	145.05 ± 21.59	144.02 ± 23.02	143.33 ± 19.23	0.897	0.800, 0.875, 0.647
DBP, mmHg	84.00 ± 12.39	83.64 ± 13.79	83.65 ± 11.18	0.982	0.880, 0.998, 0.872
Dyslipidemia, *n* (%)	26 (33.3%)	9 (21.4%)	16 (31.4%)	0.381	0.171, 0.282, 0.816
**Laboratory findings**					
Blood glucose, mmol/L	6.33 ± 2.15	5.81 ± 1.40	5.96 ± 1.54	0.269	0.137, 0.691, 0.250
HDL cholesterol, mmol/L	1.00 ± 0.31	1.05 ± 0.31	1.02 ± 0.36	0.751	0.452, 0.687, 0.731
LDL cholesterol, mmol/L	2.06 ± 0.83	2.03 ± 0.76	2.20 ± 0.88	0.555	0.874, 0.339, 0.349
TG, mmol/L	2.16 ± 1.33	1.76 ± 1.31	2.21 ± 1.30	0.199	0.112, 0.104, 0.847
TC, mmol/L	2.98 ± 1.38	3.18 ± 1.12	3.12 ± 1.59	0.720	0.451, 0.824, 0.578
Hs-CRP, mg/L^#^	2.15 (0.19–25.4)	3.48 (0.33–38.34)	1.25 (0.18–18.56)	0.167	0.397, 0.060, 0.197
**Medication**					
Pre-admission statin use (%) (>3months)	9 (11.3%)	22 (52.4%)	8 (14.8%)	<0.001	<0.001, <0.001, 0.543
Pre-admission aspirin use (%) (>3months)	3 (3.8%)	14 (33.3%)	6 (11.1%)	<0.001	<0.001, 0.008, 0.157
Pre-admission dual antiplatelet therapy use (%) (>3months)	5 (6.3%)	7 (16.7%)	1 (1.9%)	0.024	0.106, 0.020, 0.401

† *Comparison confined to between group 1 and group 2 for comparability*.

# *Medium (range); d, day; m, month*.

*BMI, body mass index; CVD, cerebro-cardiovascular disease; CAD, coronary artery disease; DM, diabetes mellitus; SBP, systolic blood pressure; DBP, diastolic blood pressure; HDL, high-density lipoprotein; LDL, low-density lipoprotein; TG, triglyceride; TC, total cholesterol; Hs-CRP, high-sensitivity C-reactive protein. NIHSS, NIH Stroke Score*.

*p^a^ values are calculated by ANOVA or chi-square test among three groups, as appropriate; p^b^ values are calculated by independent-samples t-test or Mann–Whitney U-test or chi-square test between group 1 and 2, group 2 and 3, or group 1 and 3, as appropriate*.

### Comparison of Intracranial Plaque Features Among the Three Groups

The comparison of intracranial plaque features among the three groups is shown in Table 2. Seven hundred and two intracranial plaques were identified in all participants (297 in the first-time acute stroke group; 218 in the recurrent acute stroke group, and 187 in the chronic stroke group). A mean of 4.0 ± 1.9 (median 4, range 1–9) plaques were identified per patient, with a mean of 3.7 ± 1.9 (median 3, range 1–9) plaques in anterior circulation stroke patients and 4.7 ± 1.8 (median 5, range 1–9) plaques in posterior circulation stroke patients. Recurrent acute stroke patients had more plaques than the other two groups (*p* < 0.001).

**Table 2:** Comparisons of plaque features among the three group patients.

	**Group 1, patients with first-time acute stroke (** ***N*** **= 80)**	**Group 2, patients with recurrent acute stroke (** ***N*** **= 42)**	**Group 3, patients with chronic stroke (** ***N*** **= 54)**	***p*** ^***a***^ **-value**	**Between-group** ***p*** ^**b**^ **-values: group 1 vs. 2; group 2 vs. 3, group 1 vs. 3**
**PLAQUE CHARACTERISTICS**					
Multiple plaque, *n* (%)	69 (86.2%)	39 (92.9%)	50 (92.6%)	0.371	0.376, 0.999, 0.253
Average plaque number, *n*	3.71 ± 1.96	5.19 ± 1.90	3.46 ± 1.33	<0.001	<0.001, <0.001, 0.417
Total plaque enhancement score	3.24 ± 2.12	4.29 ± 2.24	2.65 ± 1.80	0.001	0.008, <0.001, 0.106
**CULPRIT PLAQUE LOCATION**					
**Anterior circulation**					
ICA (C6-C7), *n* (%)	2 (2.5%)	3 (7.1%)	1 (1.9%)	0.956	0.388, 0.316, 0.999
ACA (A1-A2), *n* (%)	0	0	0	NA	
MCA (M1-M2), *n* (%)	55 (68.8%)	32 (76.2%)	35 (64.8%)	0.482	0.388, 0.229, 0.634
**Posterior circulation**					
VA (V4), *n* (%)	14 (17.5%)	2 (4.8%)	8 (14.8%)	0.143	0.048, 0.178, 0.681
BA, *n* (%)	9 (11.3%)	5 (11.9%)	10 (18.5%)	0.452	0.999, 0.376, 0.237
PCA (P1-P2), *n* (%)	0	0	0	NA	
**CULPRIT PLAQUE FEATURES**					
Eccentricity, *n* (%)	28 (35.0%)	18 (42.9%)	19 (35.2%)	0.660	0.395, 0.444, 0.982
Stenosis>50%, *n* (%)	71 (88.8%)	38 (90.5%)	46 (85.2%)	0.707	0.769, 0.437, 0.543
Stenosis, %	75.31 ± 18.04	80.00 ± 19.36	71.85 ± 16.09	0.087	0.169, 0.027, 0.271
Plaque length, mm	8.30 ± 4.14	8.71 ± 5.93	6.92 ± 3.73	0.112	0.632, 0.056, 0.086
Plaque thickness, mm	1.70 ± 0.74	1.70 ± 0.71	1.63 ± 0.78	0.845	0.996, 0.647, 0.590
Plaque area, mm^2^	10.68 ± 8.36	9.02 ± 7.24	9.33 ± 7.87	0.463	0.276, 0.850, 0.339
Plaque burden, %	79.10 ± 9.28	83.42 ± 6.42	74.48 ± 13.64	<0.001	0.029, <0.001, 0.012
Plaque hemorrhage, *n* (%)	8 (10.0%)	4 (9.5%)	7 (13.0%)	0.829	0.933, 0.600, 0.594
**PLAQUE ENHANCEMENT**					
Grade 0, *n* (%)	11(13.8%)	5 (11.9%)	15(27.8%)	0.060	0.774, 0.057, 0.044
Grade 1, *n* (%)	35(43.8%)	10 (23.8%)	22 (40.7%)	0.087	0.030, 0.081, 0.730
Grade 2, *n* (%)	34(42.5%)	27 (64.3%)	17 (31.5%)	0.005	0.022, 0.001, 0.198
Plaque ER	1.77 ± 0.63	2.06 ± 0.39	1.53 ± 0.37	<0.001	0.003, <0.001, 0.009
**NON-CULPRIT PLAQUE FEATURES**					
Stenosis, %	31.48 ± 19.55	34.64 ± 16.46	39.82 ± 19.74	0.046	0.382, 0.185, 0.013
Max plaque thickness, mm	1.22 ± 0.73	1.44 ± 0.62	1.27 ± 0.58	0.219	0.083, 0.238, 0.616
Enhanced plaque number, *n* (%)	118 (49.4%)	89 (50.6%)	65 (45.5%)	0.273	0.303, 0.110, 0.458
Grade 2 enhanced plaque number, *n* (%)	33 (13.8%)	18 (10.2%)	16 (11.2%)	0.636	0.414, 0.968, 0.459

*ICA, internal carotid artery; ACA, anterior cerebral artery; MCA, middle cerebral artery; VA, vertebral artery; BA, basilar artery; PCA, posterior cerebral artery, ER, enhancement ratio*.

*p^a^ values are calculated by ANOVA or chi-square test among three groups, as appropriate; p^b^ values are calculated by independent-samples t-test or Mann–Whitney U-test or chi-square test between group 1 and 2, group 2 and 3, or group 1 and 3, as appropriate*.

**Figure 2:**
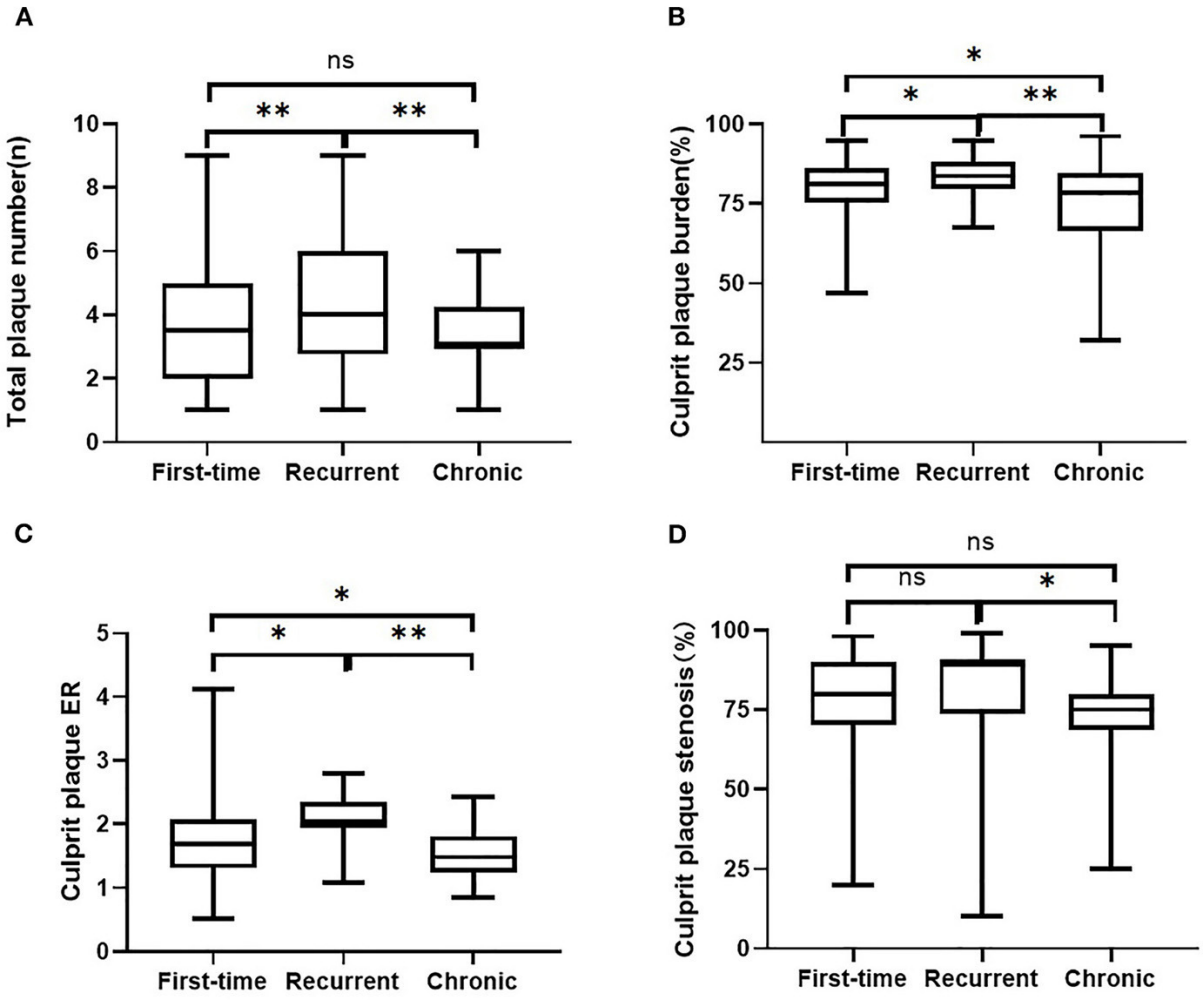
Comparisons of intracranial plaque features among the three groups. Intracranial plaque numbers **(A)** and culprit plaque features (**B**, plaque burden, **C**, enhancement ratio, **D**, degree of stenosis) on 3D MR-VWI in patient groups with the first-time acute stroke, recurrent acute stroke, and in patients with chronic stroke. ER, enhancement ratio. ^**^p < 0.001, ^*^p < 0.05, ns, no significant.

**Figure 3:**
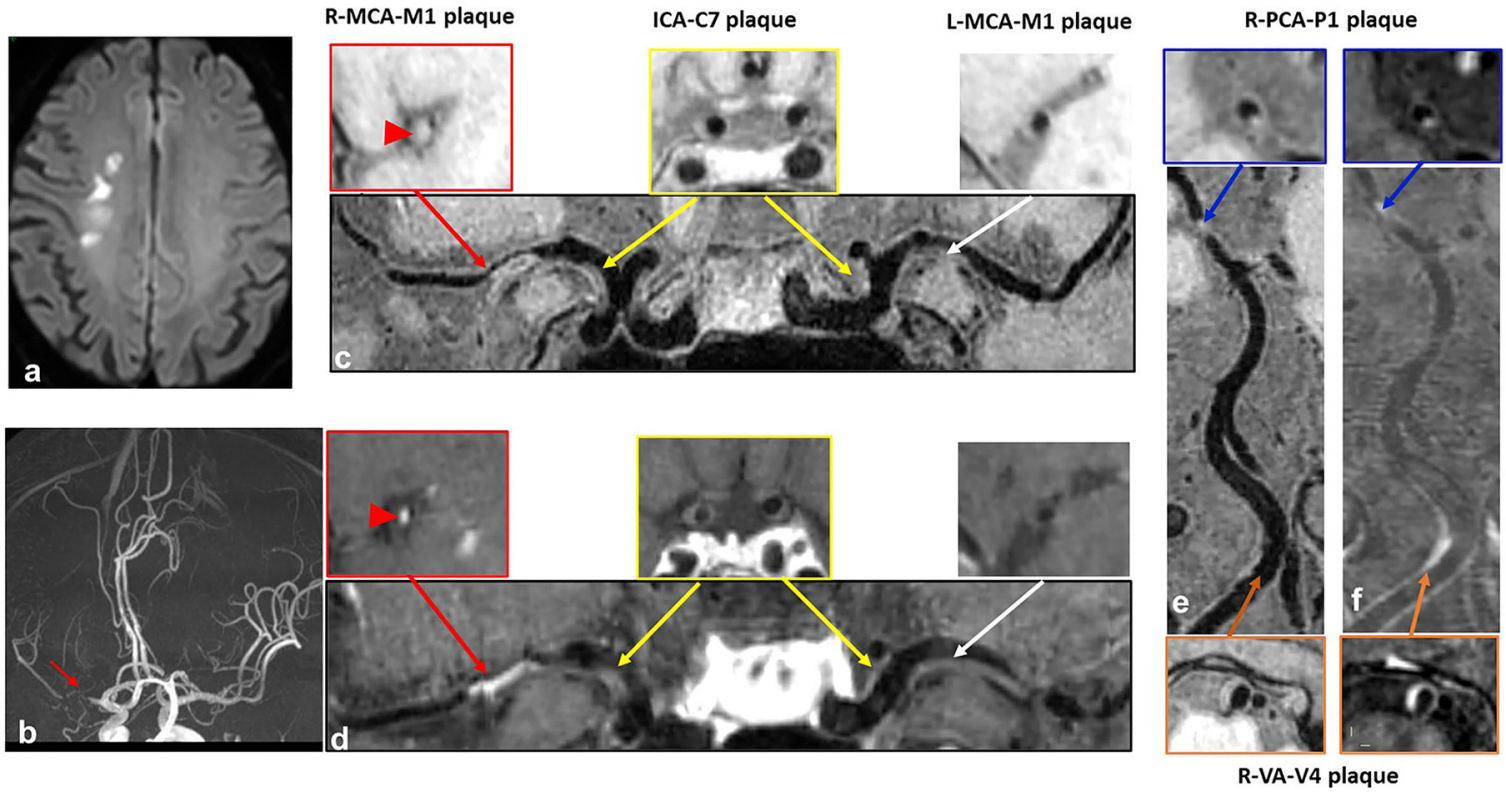
A case with recurrent acute stroke. A 62-year-old male patient presented with recurrent acute stroke. **(a)** Axial diffusion-weighted imaging detects high-signal-intensity lesions in the right corona radiata. **(b)** Time-of-flight magnetic resonance angiography image shows severe stenosis on the M1 segment of right middle cerebral artery (MCA) (red arrow) and no obvious stenosis on the left MCA, basilar artery (BA) or posterior cerebral artery (PCA). **(c–f)** curved planar reconstruction images from pre-contrast and post-contrast whole-brain vessel wall imaging show multiple plaques in the right MCA (red arrow) and left MCA (white arrow), bilateral C7 segment of internal carotid artery (ICA) (yellow arrow), P1 segment of right PCA (blue arrow), and V4 segment of vertebral artery (VA) (orange arrow). Grade 2 enhancement pattern and obvious contrast enhancement are detected in the culprit plaque of the M1 segment of right MCA (red arrowhead).

**Figure 4:**
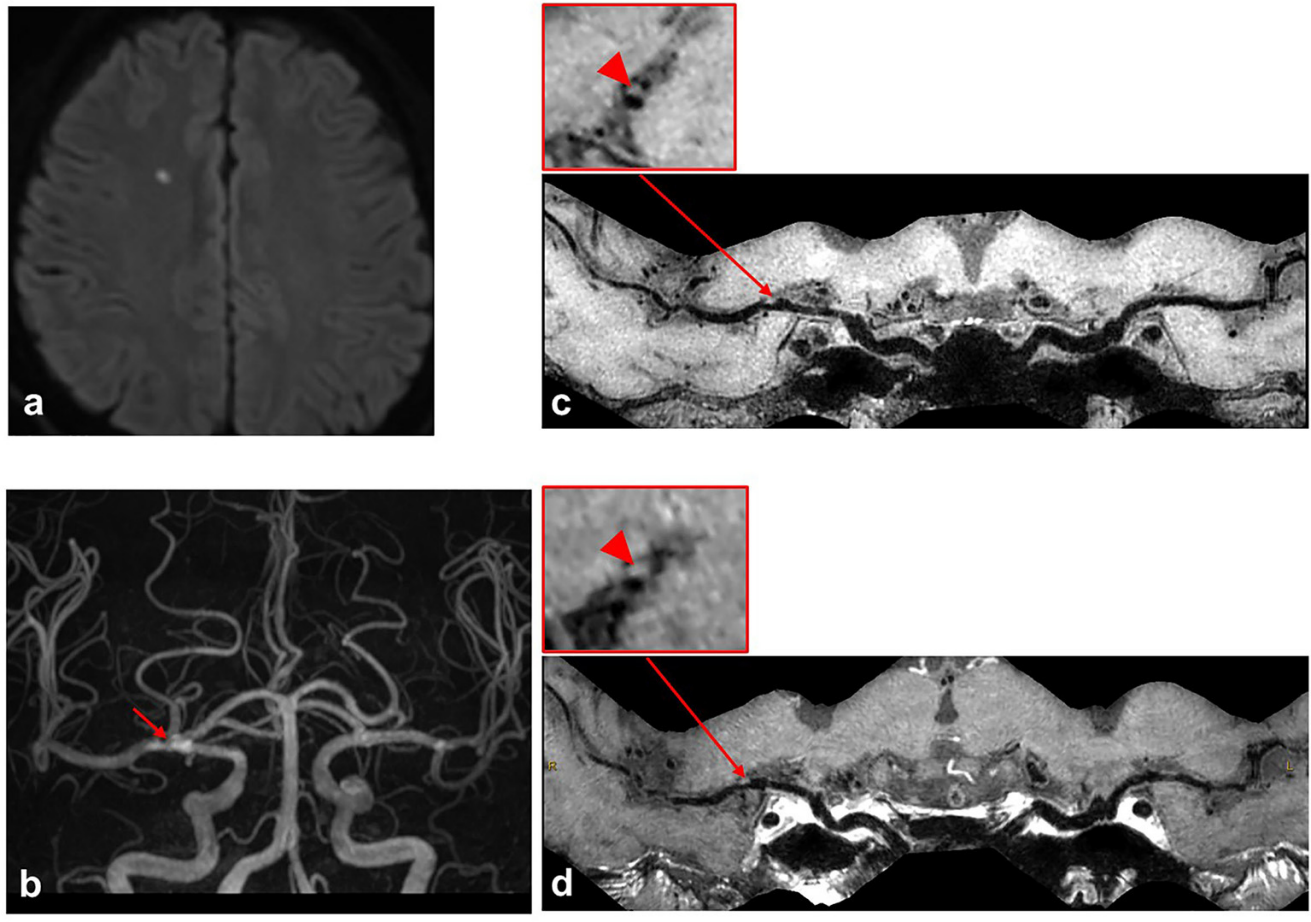
A case with first-time acute stroke. A 68-year-old male patient with the first-time acute stroke. **(a)** Axial diffusion-weighted imaging detects high-signal-intensity lesion in the right corona radiata. **(b)** Time-of-flight magnetic resonance angiography image shows moderate stenosis on the M1 segment of right middle cerebral artery (MCA) (red arrow) and no stenosis on the left MCA. **(c,d)** Curved planar reconstruction images from pre-contrast and post-contrast whole-brain vessel wall imaging show a focal plaque in the right M1 segment of MCA. No obvious contrast enhancement is identified in the culprit plaque of the right MCA M1 segment (red arrowhead).

As for culprit plaque, there was the following arterial segment involvement: 6 (3.4%) intracranial internal carotid artery, 122 (69.3%) M1-2 segment of the MCA, 24 (13.6%) V4 segment of vertebral artery, and 24 (13.6%) were found in basilar artery. Patients with acute stroke (first-time and recurrent) had significantly larger culprit plaque burden and higher plaque ER than patients with chronic stroke (*p* < 0.001, *p* < 0.001, respectively). Patients in the recurrent acute stroke group had larger culprit plaque burden (*p* < 0.001) and higher plaque ER (*p* < 0.001) than the other two groups. The degree of stenosis of culprit plaques was not significantly different among the three groups (*p* = 0.087) but was significantly different between the recurrent acute stroke group and the chronic stroke group (*p* = 0.027). Culprit plaque length (*p* = 0.112), culprit plaque thickness (*p* = 0.845), culprit plaque area (*p* = 0.463), and culprit plaque eccentricity (*p* = 0.660) were not significantly different among the three groups. The prevalence of culprit IPH was generally low in all three groups. Box plots are shown in Figure 2, and the representative cases are shown in Figures 3, 4.

No significant difference was detected in the investigated plaque features of non-culprit plaques among the three groups.

### Association Between Intracranial Plaque Characteristics and Recurrent Acute Stroke

Table 3 shows univariate and multivariate analysis results for the parameters associated with recurrent acute stroke compared with the first-time acute stroke. The VIF values between intracranial plaque enhancement score and culprit plaque ER, culprit plaque enhancement grade, total plaque number are 1.279, 1.313, and 1.258, respectively, indicating a moderate collinearity, which is unlikely to substantially affect the following multivariate analysis significantly, whereas culprit plaque ER had no collinearity with culprit plaque enhancement grade (*VIF* = 1.0) (Supplementary Table 1). After the adjustment of clinical demographic factors, CAD [odds ratio, 4.61 (95% CI, 1.11–19.18); *p* = 0.035), total plaque number [odds ratio, 1.54, (95% CI, 1.16–2.03); *p* = 0.003], culprit plaque ER [odds ratio, 2.50, (95% CI, 1.06–5.89); *p* = 0.036], and culprit plaque burden [odds ratio, 2.44, per 10% increase (95% CI, 1.24–4.79); *p* = 0.010] were all independently associated with recurrent acute stroke.

**Table 3:** Univariate and multivariate analyses for factors associated with recurrent stroke in comparison with the first-time stroke.

**Parameters**	**Univariate analysis**		**Multivariate analysis**	
	**OR (95% CI)**	***p*** **-value**	**OR (95% CI)**	***p*** **-value**
Male	0.68 (0.32–1.46)	0.323		
Age, year	1.01 (0.97–1.05)	0.517		
BMI, kg/m^2^	0.10(0.87–1.15)	0.958		
Hypertension	1.82 (0.74–4.51)	0.195		
Dyslipidemia	0.55 (0.23–1.31)	0.174		
Current smoking	1.35 (0.55–3.35)	0.512		
Alcohol use	2.09 (0.68–6.41)	0.199		
DM	1.13 (0.53–2.34)	0.761		
CAD	4.47 (1.26–15.86)	0.020	4.61 (1.11–19.18)	0.035
Hs–CRP, mg/L	1.01 (0.96–1.11)	0.429		
Culprit plaque stenosis, % ^a^	1.25 (0.96–1.53)	0.101		
Culprit plaque thickness, mm	1.00 (0.60–1.67)	0.996		
Culprit plaque length, mm	1.02 (0.94–1.10)	0.652		
Culprit plaque area, mm^2^	0.97 (0.92–1.02)	0.280		
Culprit plaque burden, % ^a^	2.03 (1.18–3.50)	0.011	2.44 (1.24–4.79)	0.010
Culprit plaque ER	2.51 (1.23–5.12)	0.011	2.50 (1.06–5.89)	0.036
Total plaque enhancement score	1.24 (1.04–1.48)	0.015	0.93 (0.72–1.19)	0.544
Total plaque number	1.47 (1.19–1.82)	<0.001	1.54 (1.16–2.03)	0.003

a *OR based on every 10% increase*.

*BMI, body mass index; CAD, coronary artery disease; DM, diabetes mellitus; Hs-CRP, high-sensitivity C-reactive protein; ER, enhancement ratio*.

**Figure 5:**
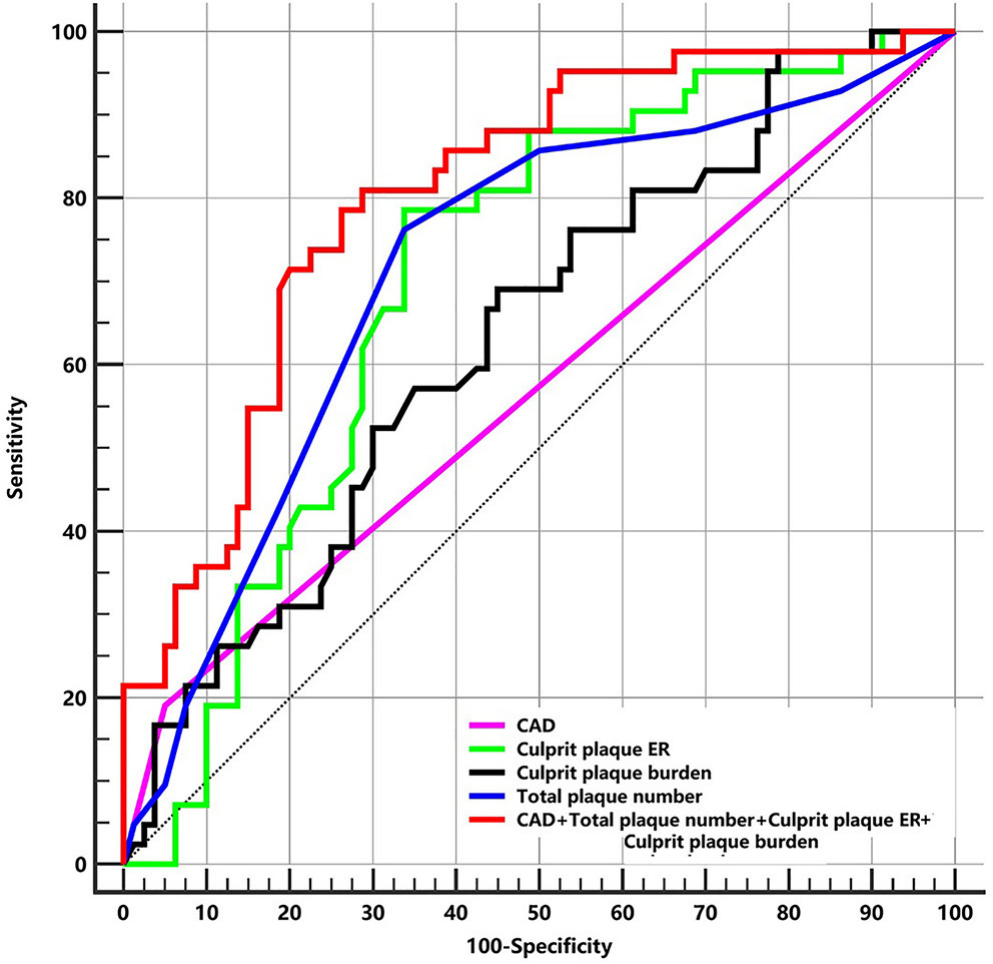
The ROC of plaque features for differentiating patients with the first-time and recurrent acute stroke. ER, enhancement ratio; CAD, coronary artery disease; ROC, receiver-operating characteristic curves; AUC, area under curve. CAD: AUC = 0.570. Culprit plaque ER: AUC = 0.704 (cutoff = 1.91, sensitivity = 78.57% specificity = 66.25%). Culprit plaque burden: AUC = 0.636 (cutoff = 81.60%, sensitivity = 69.05%, specificity = 55.0%). Total plaque number: AUC = 0.716 (cutoff = 4, sensitivity = 76.19%, specificity = 66.25%). CAD + Total plaque number + Culprit plaque ER + Culprit plaque burden: AUC = 0.804 (sensitivity = 78.57%, specificity = 73.75%).

The ROC curves of differentiating patients with the first-time and recurrent acute strokes are shown in Figure 5. The areas under the curve for CAD, culprit plaque burden, culprit plaque ER, and total plaque number were 0.570, 0.636, 0.704, and 0.716, respectively. The AUCs for CAD, culprit plaque burden, culprit plaque ER, and total plaque number were 0.570, 0.636, 0.704, and 0.716, respectively. The cutoff values of culprit plaque burden, culprit plaque ER, and total plaque number were 81.60% (sensitivity = 69.05%, specificity = 55.0%), 1.91 (sensitivity = 78.57%, specificity = 66.25%), and 4 (sensitivity = 76.19%, specificity = 66.25%), respectively. The AUC was 0.804 for the total plaque number combined with culprit plaque burden, culprit plaque ER, and CAD (sensitivity = 78.57%; specificity = 73.75%), and the corresponding cutoff values of culprit plaque burden, culprit ER, and total plaque number were 88.88%, 1.13, and 5, respectively.

The logistic regression analysis and ROC curves for differentiating between chronic and recurrent acute stroke groups were also performed in Supplementary Table 2 and Supplementary Figure 2. We found that culprit plaque ER and total plaque numbers were independently associated with recurrent acute stroke compared with chronic stroke patients (*OR* = 51.01 and *OR* = 2.30, respectively). The AUCs of culprit ER and total plaque number were 0.836 and 0.783, and a combination of the two factors achieved an excellent AUC of 0.916.

### Reliability Assessment

There were excellent intra- and inter-reader agreements for the identification of intracranial plaque, plaque enhancement grade, IPH, measurement of stenosis, plaque length, plaque thickness, plaque burden, ER, enhancement score, and total plaque number. The *kappa* values or ICC values were all higher than 0.8 (Table 4). Bland–Altman analysis results are shown in Supplementary Figure 3. Good agreement was shown on the Bland–Altman plots without significant bias. The above quantitative results of the plaque analysis were based on one reviewer (LW) because of the excellent intra- and inter-reader agreements.

**Table 4:** Inter-observer and intra-observer reproducibility (*N* = 30).

	**Inter-observer agreement**			**Intra-observer agreement**		
**Parameters**	**Bias (LOA)**	***p*** **-value**	**ICC/κ (95%CI)**	**Bias (LOA)**	***p*** **-value**	**ICC/κ (95%CI)**
Identification of intracranial plaque	–	–	0.870 (0.620–1.000)	–	–	0.870 (0.620–1.000)
Plaque enhancement grade	–	–	0.904 (0.775–1.000)	–	–	0.842 (0.674–1.000)
Intraplaque hemorrhage	–	–	1.000	–	–	1.000
Culprit plaque ER	−0.09 (−0.92–0.74)	0.456	0.839 (0.689–0.920)	0.12 (−0.66–0.90)	0.271	0.819 (0.658–0.909)
Culprit plaque enhancement score	−0.07 (−1.77–1.64)	0.893	0.897 (0.796–0.950)	0.13 (−1.77–2.04)	0.776	0.858 (0.724–0.929)
Culprit plaque length, mm	−0.18 (−2.81–2.45)	0.777	0.932 (0.863–0.967)	−0.78 (−2.88–1.33)	0.344	0.937 (0.875–0.969)
Culprit plaque stenosis, %	1.07 (−8.64–10.78)	0.453	0.977 (0.953–0.989)	3.30 (−6.19–12.79)	0.102	0.969 (0.937–0.985)
Culprit plaque burden, %	0.97 (−10.41–12.35)	0.502	0.879 (0.762–0.941)	1.62 (−10.03–13.28)	0.356	0.863 (0.735–0.932)
Culprit plaque thickness, mm	−0.04 (−0.60–0.53)	0.771	0.891 (0.784–0.947)	−0.20 (−0.83–0.43)	0.189	0.823 (0.675–0.915)
Total plaque number	0.20 (−1.88–2.28)	0.746	0.863 (0.733–0.933)	0.30 (−1.89–2.49)	0.545	0.826 (0.668–0.913)

*ICC, intraclass correlation coefficient; LoA, limitation of agreement*.

## Discussion

In this study, a group of plaque features of intracranial atherosclerotic lesions in patients with ischemic stroke was evaluated with 3D contrast-enhanced MR-VWI. We found that plaque number, culprit plaque ER, plaque burden, and CAD were independently associated with acute stroke recurrence. Our study highlights the importance of performing whole-brain vessel wall imaging and evaluating not only the culprit artery but also the entire cerebral artery tree, considering the significant associations between the total plaque number and the recurrence of acute stroke events. Different from the previous vessel wall MRI studies of recurrent ICAD stroke, the novelty of our study is that we evaluated all the arterial plaques in the Circle of Willis instead of only evaluated the features of a single culprit plaque (mostly MCA plaque or basilar artery plaque) (Kim et al., 2016; Ran et al., 2020; Shi et al., 2021). Some other studies were assessing multiple plaques in ICAD, but they didn't evaluate recurrent stroke patients (Qiao et al., 2014; Zwartbol et al., 2020). Another strength of this study is the relatively large sample size (176 patients with 702 plaques), and our results demonstrate the added value of performing 3D contrast-enhanced VWI and multiplaque analysis.

Compared with traditional 2D high-resolution contrast-enhanced MRI that was commonly used to assess intracranial plaque, 3D quantitative MRI has several major advantages. First, it covers the multiple plaques in the entire circle of Willis in a single scan, while 2D scans need multiple scans for each plaque and the slice position has inter-operator variations. Second, it provides high isotropic resolution (voxel size 0.6mm^3^ in our study); thus, the vessels can be reconstructed in any plane, while 2D imaging has larger slice thickness (2 mm mostly), which is challenging to evaluate the tortuous segments. The partial volume effect of the thick slice also reduces the accuracy when quantifying the plaque thickness, area, and plaque burden. Our study found that intracranial plaque number was independently associated with recurrent acute stroke. As a systemic disease, atherosclerosis tends to affect multiple arterial segments. Several studies have documented that multiple intracranial stenoses were associated with recurrent stroke. The Chinese Intracranial Atherosclerosis Study (CICAS) (Wang et al., 2014; Sun et al., 2018) and Clopidogrel in High-Risk Patients With Acute Nondisabling Cerebrovascular Events trial (CHANCE) (Zhu B. et al., 2018) were two multicenter, prospective MRA trials, which found that multiple ICAS was significantly associated with recurrent stroke in patients with previous stroke or transient ischemic attack. A retrospective DSA study with 576 non-disabling ischemic stroke patients established that asymptomatic ICAS was an independent risk factor for 30-days recurrent stroke (odds ratio = 2.37, 95% CI, 1.14–5.63) in patients undergoing symptomatic ICAS stenting (Zhao et al., 2018). In a prospective, long-term transcranial Doppler (TCD) follow-up study, Arenillas et al. reported that the number of coexisting asymptomatic ICAS showed a trend toward a higher recurrence rate (Arenillas et al., 2001). This supports the importance of evaluating the entire cerebral artery tree. Also, the use of 3D black blood MRI can potentially detect more plaques without stenosis than DSA (up to 27% more by a recent study) (Tian et al., 2021). Recent 3D MR-VWI studies showed that the total plaque number involving both the symptomatic intracranial and extracranial arteries had a strong predictive value for recurrent stroke (Xu et al., 2016; Li et al., 2020). Compared to previous studies, our study with 3D MR-VWI evaluated the entire cerebral vasculature out to the second-order branches, rather than only the symptomatic arterial territory; our approach enriched the existing evidence that multiple intracranial plaques are related to stroke recurrence. One potential mechanism for this finding could be that the higher atherosclerosis burden is the result of poor control of vascular risk factors that may contribute to stroke recurrence. Genetic contributors could also be a consideration, but further investigation is needed for a better understanding of the mechanisms. Besides, longitudinal studies are needed to confirm these relationships.

Previous studies showed that several intracranial plaque features were associated with recurrent stroke (Kim et al., 2016; Ran et al., 2020). Kim et al. (2016) reported that the presence of plaque enhancement predicted future stroke recurrence among stroke patients with intracranial atherosclerosis. Ran et al. (2020) found that plaque burden was associated with recurrent stroke in patients with MCA stenosis in a cross-sectional analysis comparing the first-time stroke and recurrent stroke patients. Our study showed that quantitative culprit plaque ER and plaque burden both as independent factors were associated with recurrent acute stroke, which was different from the previous studies. Disparities between our study and Kim's as compared to the study by Ran et al. could have arisen from the fact that we included both anterior and posterior circulation ICAD, while Ran et al. only analyzed symptomatic atherosclerotic MCA. Another difference between the studies was the degree of stenosis of culprit arteries. Our study included mostly patients with severe stenosis (median 80%), and the previous studies included mild–moderate stenosis patients (23.3–51.8% stenosis in Ran's study). Kim's study was longitudinal and qualitative, Ran's study was a cross-sectional and quantitative using 2D MRI, and our study was a cross-sectional and quantitative using 3D MR-VWI. The different results of these studies indicate that future larger-scale longitudinal studies with standardization of plaque measurements are needed to further establish vulnerable plaque features predicting stroke recurrence. IPH in the intracranial plaque is another culprit plaque feature that is associated with acute stroke (Xu et al., 2012; Zhu C. et al., 2018), but it hasn't been found to be associated with recurrent stroke. In our study, the prevalence of IPH was low (10.8%), which agreed with previous studies (MCA, 10.1%, BA, 17.5%) (Xu et al., 2012; Zhu C. et al., 2018). The low prevalence of IPH may be the reason why it was not significant in our study, and future larger-scale study may help better indent the role of IPH with recurrent stroke.

Previous large prospective studies showed that patients with severe symptomatic ICAS (70 to 99%) are at particularly high risk for recurrent stroke in the stenotic artery territory (5–23% at 1 year) despite standard medical therapy and control of vascular risk factors (Kasner et al., 2006; Zaidat et al., 2008; Chimowitz et al., 2011; Wang et al., 2014; Sun et al., 2018). We, however, did not find stenosis as an independent factor for recurrent stroke, possibly because our study was single-center study and all our patients had advanced atherosclerosis with acute ischemic stroke, so the stenosis range was narrow (median 80% and interquartile range, 70–90%).

Coexisting CAD is common in patients with stroke (Bae et al., 2006; Liu et al., 2020). An observational multicenter survey reported that 15.8% of patients with recent atherothrombotic cerebral infarction had a history of CAD (Leys et al., 2006). Cheng et al. found that CAD carried a 1.5-fold risk in 1-year recurrent ischemic stroke in men (Cheng et al., 2020). Man et al. (2010) reported that ischemic stroke patients with concurrent stenoses and CAD had a high risk of poor outcomes and recurrent vascular events. Our findings agree with these previous studies that CAD is associated with recurrent ischemic stroke.

Our study and most previous vessel wall MRI studies of intracranial plaque rely on manual contouring of the plaque boundaries, which is limited by the experience of readers and is time-consuming. Automatic segmentation of the intracranial vessel wall boundaries will improve the reproducibility of plaque measurements and save time. However, the segmentation of the intracranial vessel wall is a challenging task mostly due to the small size of intracranial plaque (around 2 mm thick or less), the tortuous course of intracranial arteries, and the lack of contrast between the vessel wall and surrounding tissues (Mandell et al., 2017). Therefore, the investigation of the automatic segmentation of the intracranial vessel wall is still rare. Shi et al. developed a U-net-like fully convolutional network (FCN) method to automate vessel wall segmentation in a cohort of 56 patients with intracranial plaque and achieve a Dice coefficient of 0.77 for vessel wall segmentation (Shi et al., 2019). With the strong efforts of the research community on developing methods in intracranial vessel wall segmentation, and the continuous improvement in imaging techniques with novel sequences and ultra-high-field strength (Zhu et al., 2016), more robust and automatic techniques will be available for clinical applications soon.

This study has several limitations. First, this is a cross-sectional study, and all MRIs were performed after the ischemic stroke event. The plaque morphology may change after stroke. Therefore, our results could only demonstrate the possible association between plaque features and recurrent acute stroke, but not the causality. Our results should be interpreted with caution, and our findings require a validation with future larger-scale longitudinal studies. Second, we did not evaluate collateral circulation and concurrent extracranial stenosis, both of which might play an important role in the assessment of cerebral hemodynamics and stroke risk. Future development of a risk stratification model incorporating ICAD burden, concurrent extracranial stenosis, and collateral status could provide a valuable tool for assessing the risk of recurrent ischemic events. Third, although we excluded patients with strong flow artifacts that affect the evaluation of the plaque features, there were still some moderate flow artifacts that existed. Additional blood suppression techniques, including MSDE (Zhu et al., 2014) and DANTE (Zhu et al., 2019), should be used in future studies to reduce the flow artifacts and improve the image quality. Fourth, the exact mechanism accounting for intracranial plaque enhancement remains unknown due to the lack of histology validation, and its relationship with intracranial plaque inflammation is not well-understood. Future studies using contrast agents directly targeting inflammation [like ultra-small superparamagnetic iron oxides (USPIOs)] (Hope et al., 2015) will allow the evaluation of vessel wall inflammation directly. And quantitative 3D analysis methods, including radiomics (Shi et al., 2018), will improve the evaluation of contrast enhancement.

## Conclusion

Increased intracranial atherosclerotic plaque number, higher culprit plaque ER, greater culprit plaque burden, and CAD are independently associated with recurrent acute stroke.

## Data Availability Statement

The raw data supporting the conclusions of this article will be made available by the authors, without undue reservation.

## Ethics Statement

The studies involving human participants were reviewed and approved by Research Ethics Committee of Renji Hospital, School of Medicine, Shanghai Jiaotong University. The patients/participants provided their written informed consent to participate in this study.

## Author Contributions

LW, XLi, and JinZ performed the MR examination. BS, LW, and JiaZ performed the image analysis. BS and LW performed the statistical analysis. CZ and HZ participated in the study design. YZ, XLiu, and JX reviewed the manuscript. BS was a major contributor in writing the manuscript. All authors read and approved the final manuscript, contributed to the discussion, read, and approved the final manuscript.

## Conflict of Interest

The authors declare that the research was conducted in the absence of any commercial or financial relationships that could be construed as a potential conflict of interest.

## Publisher's Note

All claims expressed in this article are solely those of the authors and do not necessarily represent those of their affiliated organizations, or those of the publisher, the editors and the reviewers. Any product that may be evaluated in this article, or claim that may be made by its manufacturer, is not guaranteed or endorsed by the publisher.

## Abbreviations

ACA: anterior cerebral artery
BMI: body mass index
BA: basilar artery
CVD: cerebro-cardiovascular disease
CAD: coronary artery disease
DBP: diastolic blood pressure
DM: diabetes mellitus
DWI: diffusion-weighted imaging
ER: enhancement ratio
FLAIR: fluid-attenuated inversion recovery imaging
HDL: high-density lipoprotein
Hs-CRP: high-sensitivity C-reactive protein
ICAD: intracranial atherosclerosis disease
ICA: internal carotid artery
LDL: low-density lipoprotein
MCA: middle cerebral artery
MR-VWI: MR vessel wall imaging
NIHSS: NIH Stroke Score
PCA: posterior cerebral artery
SBP: systolic blood pressure
TG: triglyceride
TC: total cholesterol
T1-VISTA: T1WI volume isotropic turbo spin-echo acquisition
VA: vertebral artery.
