# Supplementary material for: Intracranial Atherosclerotic Plaque Characteristics and Burden Associated With Recurrent Acute Stroke: A 3D Quantitative Vessel Wall MRI Study
Source: Front Aging Neurosci. 2021 Jul 28;13:706544. doi: 10.3389/fnagi.2021.706544 (PMC8355600; doi:10.3389/fnagi.2021.706544)
Supplement: Supplementary file 1 [file Data_Sheet_1.pdf]

Supplemental Table 1. The multicollinearity diagnosis of the independent variables in the regression model.

	Total enhancement score
**Independent variables**	**VIF**
Culprit plaque enhancement ratio	1.279
Culprit plaque enhancement grade	1.313
Total plaque number	1.258
	**Culprit plaque enhancement ratio**
**Independent variables**	**VIF**
Culprit plaque enhancement grade	1.000

VIF: variance inflation factor

Supplemental Table 2. Univariate and multivariate analysis for factors associated with recurrent acute stroke in comparison to chronic stroke.

Parameters	Univariate analysis		Multivariate analysis	
	OR (95%CI)	p value	OR (95%CI)	p value
Male	0.92(0.40-2.018	0.835		
Age, year	0.97(0.93-1.01)	0.172		
BMI, kg/m^2	0.98(0.82-1.17)	0.823		
Hypertension	1.21 (0.45-3.31)	0.704		
Dyslipidemia	0.65 (0.25-1.66)	0.365		
Current smoking	2.10(0.72-6.09)	0.173		
Alcohol use	3.40(0.82-14.06)	0.091		
DM	1.78 (0.77-4.15)	0.181		
CAD	1.58 (0.52-4.78)	0.418		
Hs-CRP, mg/L	1.09(0.98-1.22)	0.101		
Culprit plaque stenosis, % [a]	1.33(1.02-1.74)	0.033		
Culprit plaque thickness, mm	1.14(0.66-1.96)	0.645		
Culprit plaque length, mm	1.08 (0.99-1.19)	0.085		
Culprit plaque area, mm^2	0.99(0.94-1.05)	0.841		
Culprit plaque burden, % [a]	2.50 (1.46-4.27)	<0.001		
Culprit plaque ER	30.18(7.51-121.24)	<0.001	51.01(9.50-273.96)	<0.001
Total plaque enhancement score	1.49(1.19-1.86)	<0.001		
Total plaque number	1.97(1.43-2.70)	<0.001	2.30(1.51-3.50)	<0.001

a: OR based on every 10% increase. BMI, body mass index; CAD, coronary artery disease; DM, diabetes mellitus; Hs-CRP, High-sensitivity C-reactive protein; ER, enhancement ratio.

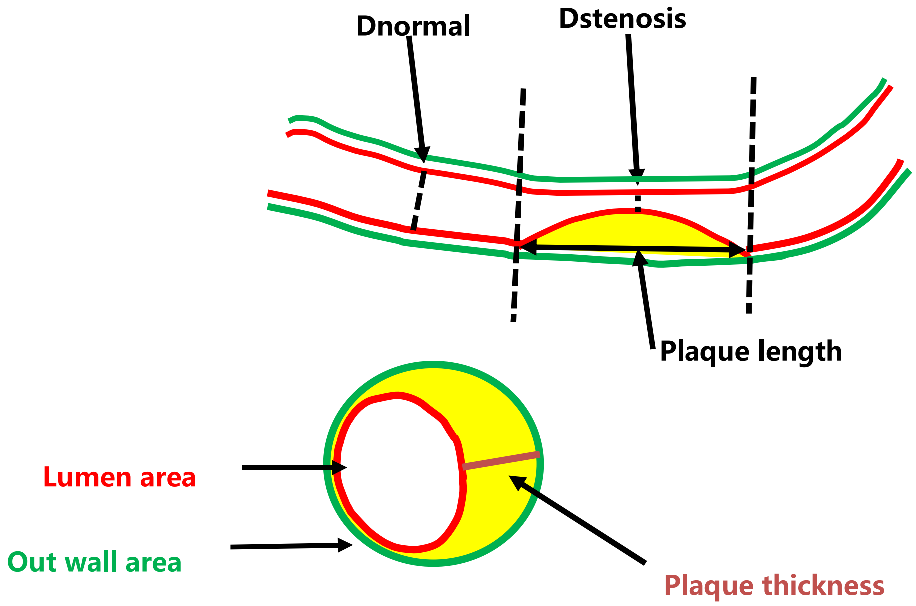

Supplemental Figure 1. Diagram for the measurements of plaque morphology.

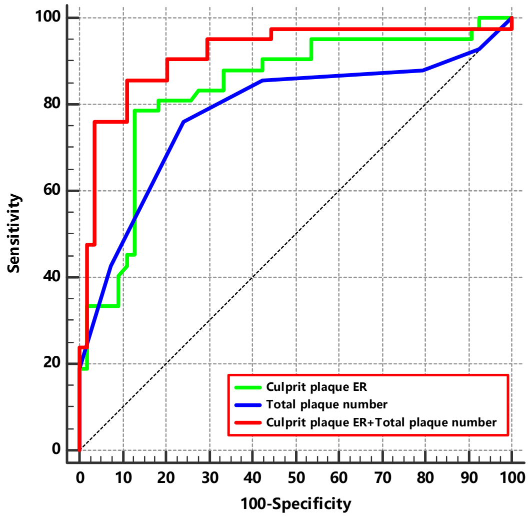

Supplemental Figure 2. The receiver operating characteristic curves of plaque features for differentiating patients with recurrent acute stroke and chronic stroke.

ER, enhancement ratio.

Culprit plaque ER: AUC=0.836 (cutoff=1.84, sensitivity=80.95% specificity=81.48%)

Total plaque number: AUC=0.783 (cutoff=4, sensitivity =76.19%, specificity =75.93%)

Culprit plaque ER + Total plaque number: AUC=0.916 (sensitivity =85.71%, specificity =88.89%)

a

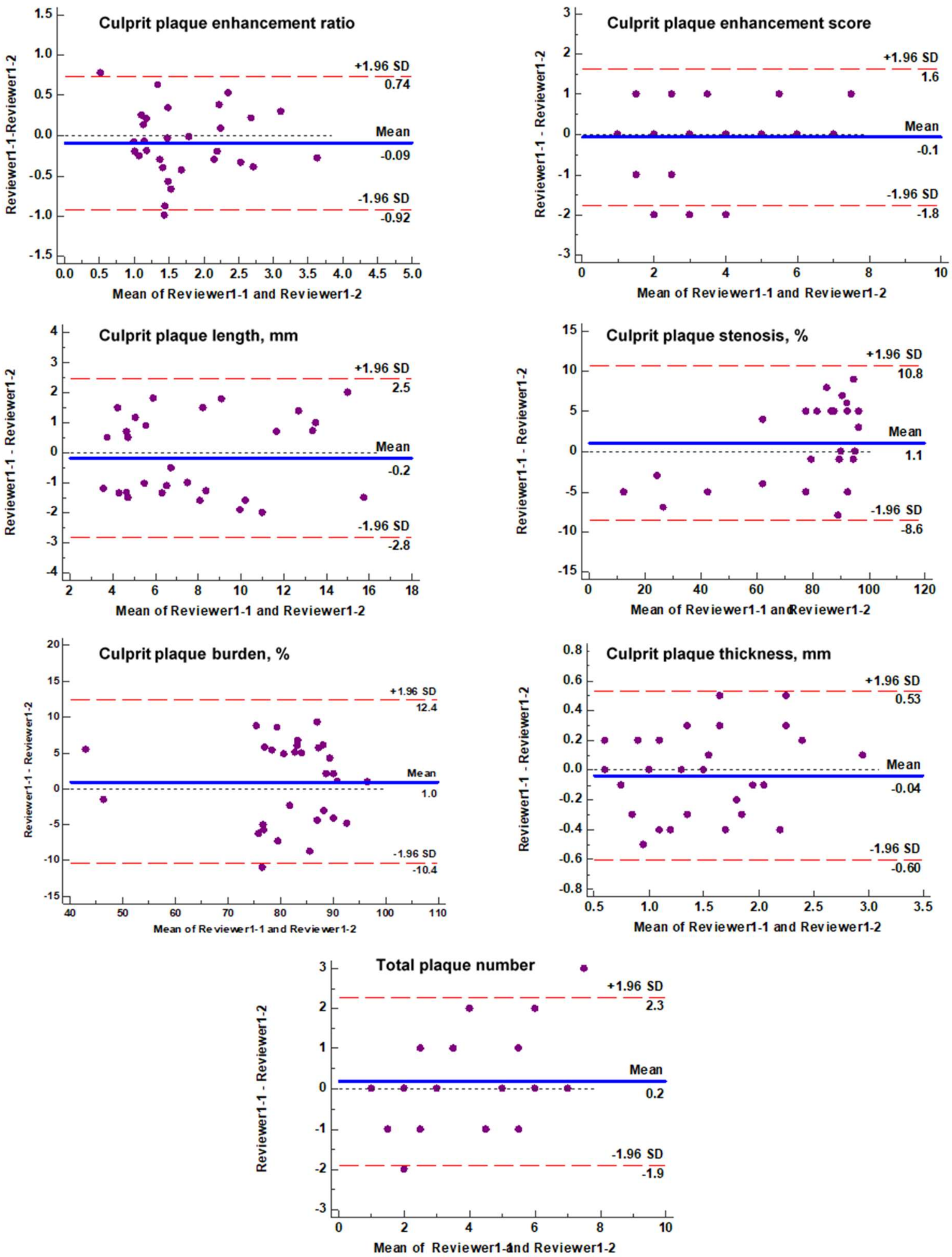
Culprit plaque enhancement ratio
Reviewer1-1-Reviewer1-2
Mean of Reviewer1-1 and Reviewer1-2
+1.96 SD
0.74
Mean
-0.09
-1.96 SD
-0.92
Culprit plaque enhancement score
Reviewer1-1 - Reviewer1-2
Mean of Reviewer1-1 and Reviewer1-2
+1.96 SD
1.6
Mean
-0.1
-1.96 SD
-1.8
Culprit plaque length, mm
Reviewer1-1 - Reviewer1-2
Mean of Reviewer1-1 and Reviewer1-2
+1.96 SD
2.5
Mean
-0.2
-1.96 SD
-2.8
Culprit plaque stenosis, %
Reviewer1-1 - Reviewer1-2
Mean of Reviewer1-1 and Reviewer1-2
+1.96 SD
10.8
Mean
1.1
-1.96 SD
-8.6
Culprit plaque burden, %
Reviewer1-1 - Reviewer1-2
Mean of Reviewer1-1 and Reviewer1-2
+1.96 SD
12.4
Mean
1.0
-1.96 SD
-10.4
Culprit plaque thickness, mm
Reviewer1-1 - Reviewer1-2
Mean of Reviewer1-1 and Reviewer1-2
+1.96 SD
0.53
Mean
-0.04
-1.96 SD
-0.60
Total plaque number
Reviewer1-1 - Reviewer1-2
Mean of Reviewer1-1 and Reviewer1-2
+1.96 SD
2.3
Mean
0.2
-1.96 SD
-1.9

b

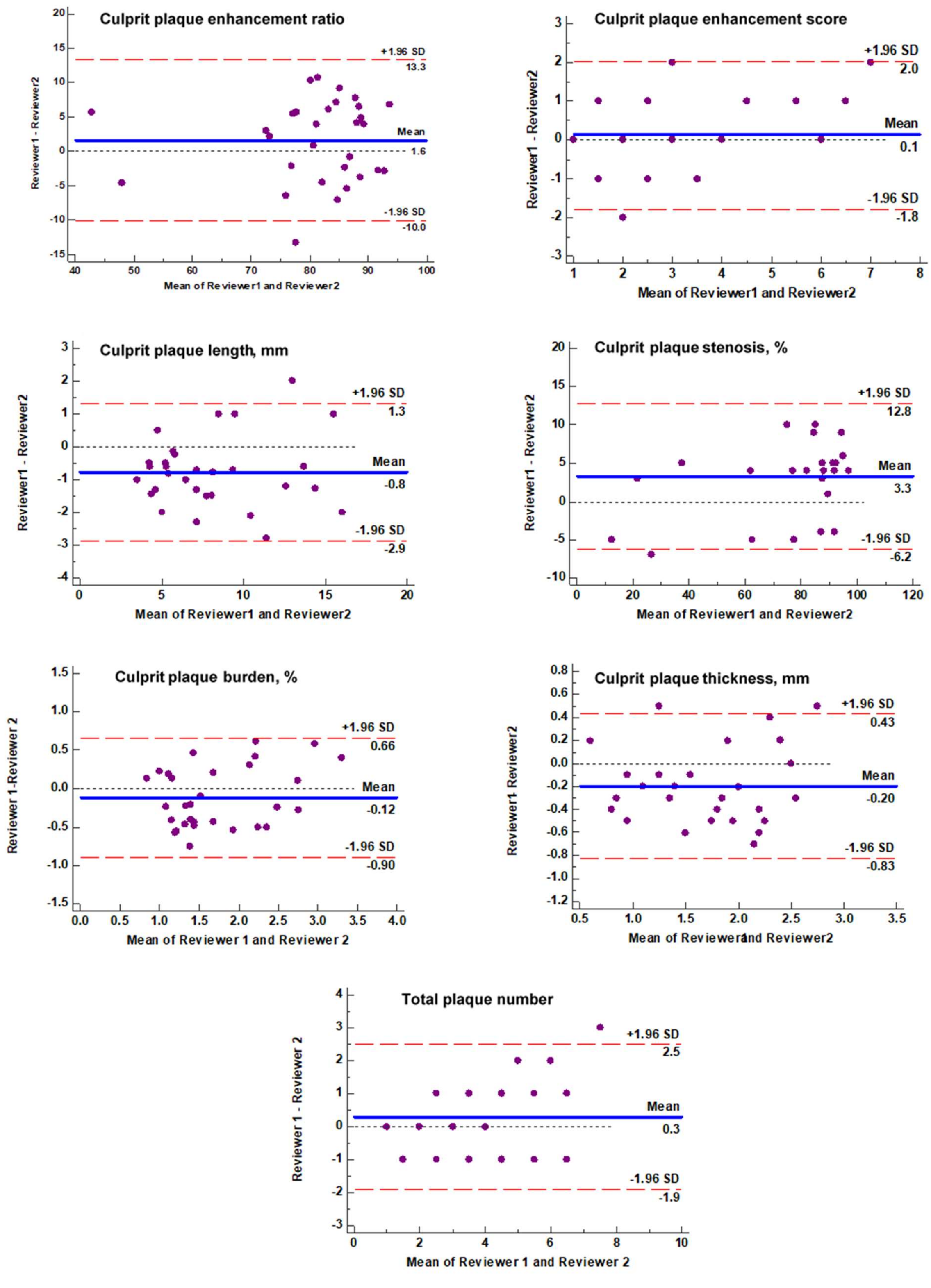

Supplemental Figure 3. Inter (a) and intra-observer (b) agreement of continuous data by Bland-Altman analysis.
